# If I was minister of health I would disable ‘customer service’ reviews on the NHS website

**DOI:** 10.1177/0141076820975366

**Published:** 2020-12-17

**Authors:** Olivia Holtermann Entwistle

**Affiliations:** Division of medicine, Imperial College London, London SW7 2AZ, UK; London North West University Healthcare Trust, Division of Medicine, Watford Road Harrow HA1 3UJ

‘All staff are polite and well dressed with good customer services always helped out whenever’ *– Anonymous, NHS Choices*^1^

Having worked as an NHS junior doctor for 12 months, during perhaps the most tumultuous period of its history, I have seen first-hand how a lack of resources and high demand are stretching the health service. However, the precarious position the NHS currently finds itself in is as much a product of a political project which has sought to marketise our relationship to healthcare, as it is of insufficient funding. This ideological paradigm – distilled in the TripAdvisor style review function on the NHS website – recasts patients as consumers and public goods as market products. It is also these consumer logics that are so often marshalled to justify cuts and privatisation in the name of ‘efficiency’ and ‘customer service’.

From the short time I have spent working as a doctor, I am already certain that the clinician–patient relationship is irreducible to that of producer–consumer, and that attempts to shoehorn healthcare into the mould of consumerism has deleterious effects on patients and healthcare workers. As Minister of Health, I would therefore seek to turn back the tide of consumerism in healthcare by disabling the online ‘customer review’ function on the NHS website, while maintaining robust and transparent complaints procedures.

Now is the moment to take such a much-needed step. The COVID-19 crisis has illustrated that the NHS simply does not exist in the public imagination as a consumer service and that it has always been understood first and foremost as a collectively owned public good that helps us all in our hour of need. Nigel Lawson was not so wide of the mark in his somewhat snide assertion that the NHS is the closest thing the English have to a religion.[Bibr bibr2-0141076820975366]

## From patients to consumers in British healthcare

Prior to the establishment of the NHS, British patients largely accessed healthcare in the mode of consumers, seeking out services in the marketplace and paying directly for them.^2^ Although there was some social provision, many found themselves excluded from healthcare.^3^ With the establishment of the NHS, the link between financial means and access to healthcare was severed.^4,5^ Citizens became both patients and stakeholders in a system that promised free healthcare at the point of use from the ‘cradle to the grave’. However, the NHS still operated within a medical culture in which clinicians could be paternalistic, and patients often had little control over their own health. In response to this, consumer groups such as the Patients Association became centres of advocacy for patients' rights and lobbied for improvements, from access to medical records to transparent complaints procedures.^6^

With the arrival of Thatcher, the manifestation of consumerism in healthcare shifted from emphasising patients' rights and autonomy to a sustained attempt to transmute the patient into a consumer, defined in simple terms as someone who exercises choice in a marketplace.^7^ New Labour similarly took up the mantle of consumerism in an effort to mirror what they viewed as a widespread ‘consumer culture’.^6,7^ Public services, however, were seen as insufficiently sensitive to people's wants and thus unfit for the 20th-century consumer who expected to have freedom to pursue their individual desires.^7^ Foregrounding consumer freedom, they argued, would have the dual effect of generating greater individual satisfaction and driving NHS improvement.^8^ Introducing choice in public services therefore became the focus of New Labour's reform agenda, an ideological project deepened by the Coalition Government and Cameron's Conservative administration,^9^ who oversaw the introduction of online reviews.^10^

Arguably however, these reforms were not only *responding* to consumer culture but also sought to further it, folding consumer narratives into ever more areas of life and reconstructing the citizen–patient as customer. Online reviews, in particular, impose consumer practices upon the NHS, instructing patients to relate to the system in the transactional mode of the customer, and encouraging people to view the service as they would a restaurant or hotel.

## Not quite patients, not quite consumers: the inherent incompatibility of healthcare and consumerism

Despite the ‘political–cultural work’ (Clarke,^7^ p. 239) done to recast the British patient as a consumer, socialised healthcare is an area of life peculiarly resistant to being subsumed by market narratives. Both practical constraints and the emotional relationship that defines a person's interactions with healthcare make this transition impossible. This tussle creates dislocated patients and dissatisfied clinicians who find themselves at the rupture point between the market and the welfare state.

At their most general, online reviews invite patients to locate the locus of flawed services at the level of the individual healthcare worker, practice or hospital. Rating and reviewing a general practice for ‘customer service’ does not take account of both local and national conditions in which it operates. Unlike formal evaluation by the Care Quality Commission or systematised and transparent complaints and feedback procedures, online reviews do little to engender systemic change and, in the case of failings, serve only to individualise responsibility.

At the level of the individual interaction with healthcare, if we take the consumer to be defined by the ability to choose, then a socialised healthcare system with limited resources will inevitably frustrate the freewheeling consumer. The NHS simply does not have the resource capacity to provide a wide array of choices.^11^ Even in areas where patients nominally have a choice, such as primary care, the range of options is small. In the case of general practices, for example, patients are free to choose, but usually only within their catchment area. This ‘choice’ then becomes akin to a ‘like it or lump it’ decision, creating frustrated consumers who are denied the level of freedom they have been instructed to seek.^7^

In the encounter between patient and doctor, this inconsistency is made yet more obvious. Not only does the ‘consumer’ suffer a lack of choice in where or by whom they are cared for, they may also find their decisions about specific investigations or treatments curtailed.^12^ The clinician's primary motivation is to address clinical needs, grounded in knowledge of the patient's condition, and to formulate a management plan in line with the individual's ‘best interests’. The potential disparity in knowledge and understanding about health between the patient–consumer and doctor may generate a different understanding of needs.^11,12^ What the patient *needs* may not be what the patient *wants*. But, for the consumer, want and need become interchangeable. As clinicians, however, we cannot subordinate clinical needs to patient wants when the two are incompatible. This would, in fact, be counter to a doctor's professional commitments as laid out by the General Medical Council.

Furthermore, NHS clinicians function within a system in which the needs of many must be accounted for. We expect clinicians to act not only as providers of services to the patient before them but also as stewards of the system *as a whole*. If, however, we view consultations as exchanges between doctors and consumers, then concern for the general population or even the next patient would be a pollutant. The consumer narrative of individual choice thus butts uncomfortably against the realities of a tightly resourced system designed to provide healthcare for many. It is no surprise that at my medical school a common practical examination scenario was explaining to a patient why they could not have an MRI for back pain. The patient–consumer *wants* but does not need this scan. And yet, if he were a true consumer he could indeed demand it. In the absence of such a reality, it becomes the individual clinician who is seen to obstruct consumer choice, thus threatening to establish an adversarial relationship between doctor and patient. In short, asking people to view themselves as consumers and thrusting them into a system unable to reliably take account of their ‘consumer’ status does them a disservice. And to ask clinicians to straddle this cultural rupture point is equally to expect too much.

Nor are clinicians themselves impervious to consumer culture, despite their thorny interactions with it. Although I doubt many clinicians view their patients as customers, organisations as a whole cannot avoid the pull towards foregrounding ‘customer service’ once it becomes the terms by which funding is awarded and performance measured. For example, an article on Practice Index advises general practitioners on how to improve their online reviews, noting that such reviews have a measurable impact on the number of new patients a practice attracts.^13^ Funding is awarded based on the number of patients on a practice's list,^14^ thus incentivising the pursuit of better reviews and, as consumer logic would have it, the consumer is satisfied when their desires are met. Yet, as we have seen, seeking to meet desires alone is neither ethically nor financially viable.[Bibr bibr11-0141076820975366] The inability to square this circle has left the online review system open to abuse, with investigations finding many reviews to be fakes produced by healthcare workers themselves.^15^ This fact both calls into question the robustness of online reviews and demonstrates the ways in which they may have a corrupting effect on those working within the system.

Notwithstanding, and at the crux of the issue, is that consumerism is fundamentally inconsistent with how patients view themselves, and fails to recognise that the need to access healthcare is not a ‘consumer choice’ but one often thrust upon an individual in times of distress.^12^ The ‘services’ of healthcare are far more than simply testing and treatment: care entails human relationships that offer companionship and collaboration in facing the fears and uncertainties inherent in illness. Research carried out by Clarke and Newman found that only 6 in 97 people accessing NHS services viewed themselves as a ‘consumer’ or ‘customer’ and their accompanying answers indicate how the customer–provider paradigm fails to capture the complexity of people's interactions with healthcare: ‘I feel involved in my case … this relationship – doctor/patient – is right for me, and I feel more than a consumer or customer’ (Clarke and Newman,^16^ p. 746). Likewise, I am certain my patients would feel offended if they found I viewed them as a customer, or that my motivation was grounded in seeking a positive review. The episodic and transactional encounter with a service entailed by the consumer paradigm does not reflect the relationships patients and clinicians form, in which decisions and burdens alike are shared. As one person said during their interview, ‘as a patient I am part of a team and care works both ways’ (Clarke and Newman,^16^ p. 747).

## ‘Our NHS’: the health service in the COVID-19 era

If we recognise the incongruity of consumerism with a nationalised healthcare system, this incongruity appears even starker through the lens of the COVID-19 pandemic, which has restated the public's deep attachment to the NHS. The last few months have seen an outpouring of gratitude and even love towards the health service, accompanied by a reaffirmation of the NHS as a collectively owned public good. It is no longer *The NHS*, it is *Our NHS*.

Thus, we find ourselves at a junctural moment in our relationship to the health service. One we can seize to reframe ourselves as patients and clinicians working in collaboration for both the individual and collective good. In addition, it forces us all to recognise that health at a population level requires collective behaviour; not only do we all have a stake in the NHS, we all have a stake in generating health for ourselves and those around us.

Many predictions about the transformative effect of this crisis have no doubt been overstated. However, COVID-19 has undeniably destabilised the notion of the patient as customer which neither reflects the national mood nor the experience of patients and clinicians. Making the modest adjustment of disabling the ‘customer review’ function on the NHS website would, I believe, cement this shift, moving us towards a new cultural landscape underpinned by a recognition that the NHS is, and always has been, a collective project.

## Declarations

**Ethics approval:** Not applicable.

**Guarantor:** OHE.

**Contributorship:** Sole author.
